# Mammographic Calcifications in Lung Transplant Recipients: Prevalence and Evolution

**DOI:** 10.3390/biomedicines13092318

**Published:** 2025-09-22

**Authors:** Jonathan Saenger, Jasmin Happe, Caroline Maier, Bjarne Kerber, Ela Uenal, Denise Bos, Thomas Frauenfelder, Andreas Boss

**Affiliations:** 1 Diagnostic and Interventional Radiology, University Hospital Zurich, University Zurich, 8091 Zurich, Switzerland; 2 Institute of Diagnostic and Interventional Radiology, GZO Regional Health Center, 8620 Wetzikon, Switzerland; 3 Institute of Diagnostic and Interventional Radiology and Neuroradiology, University Hospital Essen, University Duisburg-Essen, 45147 Essen, Germany

**Keywords:** lung transplantation, breast cancer, screening, mammography

## Abstract

**Objective:** To investigate the prevalence and progression of macrocalcifications or sporadic scattered microcalcifications, breast arterial calcifications (BAC) and grouped microcalcifications in women undergoing lung transplantation (LTX). **Materials and Methods:** In this retrospective single-center cohort study, 176 adult female patients who underwent mammography between 2008 and 2025 were included: 82 LTX recipients and 94 age-matched controls. Mammographic findings were assessed using standardized BI-RADS criteria and a visual BAC scoring system. Clinical and demographic data were extracted from electronic medical records. Multivariable logistic regression and cumulative incidence analysis were used to evaluate associations and progression patterns. Interobserver agreement was assessed using Fleiss’ kappa. **Results:** BAC and grouped microcalcifications were significantly more prevalent in the LTX group in the last mammography (BAC: OR 6.57, 95% CI 2.34–20.7; microcalcifications: OR 14.6, 95% CI 3.93–73.9; both *p* < 0.001). Cumulative incidence analysis showed accelerated progression of BAC and grouped microcalcifications in LTX recipients (*p* ≤ 0.01), while macrocalcifications or sporadic scattered microcalcification progression did not differ significantly. BAC was often more extensive and potentially mimicked malignant findings. Interobserver agreement was highest for the four-level BAC scoring system (κ = 0.61), followed by BAC presence (κ = 0.59) and macrocalcifications (κ = 0.51), while grouped microcalcifications showed only fair agreement (κ = 0.33). **Conclusions:** Lung transplant recipients demonstrate significantly higher prevalence and faster progression of BAC and grouped microcalcifications compared to controls, complicating mammographic interpretation. Given their elevated risk of aggressive malignancies and diagnostic overlap between benign and suspicious calcifications, transplant recipients may benefit from tailored screening strategies.

## 1. Introduction

Breast cancer remains one of the leading causes of cancer-related mortality among women worldwide [1]. Early detection through mammography screening has significantly improved outcomes by identifying malignancies at a treatable stage, thereby reducing the need for radical surgery and improving survival rates [2,3]. In solid organ transplant recipients, including those undergoing lung transplantation (LTX), breast cancer represents a distinct clinical challenge [4]. Although breast cancer incidence overall appears comparable to that of the general population, outcomes are generally worse, with transplant recipients being diagnosed at younger ages, presenting with more aggressive tumor biology, and experiencing higher cancer-related mortality [5,6,7]. These adverse outcomes are likely due to immunosuppressive therapy, altered immune surveillance, and delayed diagnoses due to overlapping comorbidities [8,9].

In this high-risk population, mammography remains the standard screening tool [4] A key radiologic feature in breast cancer detection is the presence of microcalcifications, which are seen in up to 40% of invasive cancers and are essential for identifying the majority of ductal carcinoma in situ (DCIS) [3,10,11,12]. However, not all calcifications are malignant. Benign forms, such as coarse or dystrophic macrocalcifications, are frequently observed and may coexist with suspicious findings, potentially obscuring subtle malignant features or complicating interpretation [13]. These macrocalcifications can result from benign processes including fat necrosis, involutional changes, or post-surgical scarring [13]. Breast arterial calcifications (BACs) represent another benign entity, typically appearing as linear or tubular “railroad track” patterns along vascular structures [14,15,16]. BACs are associated with age, diabetes, chronic kidney disease, and other cardiovascular risk factors [17,18,19]. While considered non-cancerous, BAC can mimic malignant calcifications, especially when fragmented or atypically distributed, thereby posing a diagnostic challenge [20]. This may be particularly pronounced in LTX recipients, who are prone to vascular remodeling, systemic inflammation, and metabolic disturbances [7,21]. These factors may influence the prevalence, morphology, and progression of calcifications, further complicating radiologic interpretation.

Despite growing recognition of BAC as a potentially meaningful mammographic finding, little is known about its prevalence and evolution in lung transplant recipients. Moreover, the extent to which BAC may overlap with or obscure grouped microcalcifications in this vulnerable group has not been evaluated. This study aims to (i) assess the prevalence and progression of benign calcifications, including macrocalcifications or sporadic scattered microcalcifications, and BAC, as well as potentially suspicious grouped microcalcifications in lung transplant recipients compared to matched controls, and (ii) examine possible diagnostic overlap that may influence the accuracy and interpretation of breast cancer screening.

## 2. Materials and Methods

### 2.1. Patient Selection

The local ethics committee approved this retrospective study, with all procedures conducted under institutional policies, federal regulations, and the Declaration of Helsinki. The cohort included consecutive adult female patients who underwent mammography at our institution between February 2008 and May 2025. Patients with more than two sequential post-transplant mammographies available were identified through the institutional Picture Archiving and Communication System (PACS). A control group of screening patients with more than two sequential mammographies was included for comparison. Patients with concurrent transplantations, such as kidney or liver, were excluded. Clinical data, including relevant comorbidities such as chronic kidney disease, diabetes mellitus, and pulmonary hypertension or hypertension, were systematically extracted from electronic medical records. As medical information was not consistently available for all time points, prevalence was calculated based on the most recent available report, and it was assumed that documented medical comorbidities were present throughout the entire observation period.

### 2.2. Mammography Protocol

Mammograms were performed following the breast cancer screening guidelines of the American College of Radiology at the University Hospital of Zurich or affiliated outpatient clinics in the Zurich region, Switzerland. Standard craniocaudal and mediolateral oblique views were acquired for each breast [22]. Digital breast tomosynthesis was introduced at our institution in 2022 and was available only for a limited subset of patients; to maintain consistency across the cohort, it was not included in the present analysis. Mammography was conducted using one of the following systems: Senographe Essential Acquisition System ADS 56.21.3 (General Electric, Boston, MA, USA), Image SDL or Class System (Internazionale Medico Scientifica/Giotto, Sasso Marconi, Italy), Selenia Dimensions (Hologic, Marlborough, MA, USA), or Mammomat Novation DR/Mammomat Inspiration System (Siemens Healthcare GmbH, Forchheim, Germany).

### 2.3. Mammographic Assessment

Breast density, BI-RADS classification, and the presence of calcifications were systematically assessed by the fifth edition of the BI-RADS guidelines. Macrocalcifications were defined as coarse calcifications larger than 0.5 mm, often appearing as round, dystrophic, or vascular. Grouped microcalcifications were defined as five or more fine calcifications clustered within a 1 cm^3^ area, which may raise suspicion depending on their morphology and distribution. BAC was defined as linear or tubular calcifications following the typical course of vessels, often described as parallel “railroad track” patterns [18]. To assess the extent of BAC, a structured visual scoring system as proposed by Saenger et al. was adapted. The scoring system included four grades: BAC Grade 1 was defined as the absence of vascular calcifications, with only macrocalcifications or sporadic, scattered microcalcifications present. BAC Grade 2 was defined by a few discontinuous calcified particles seen in a single area with questionable association to a tubular structure, resembling fibroglandular microcalcifications. Calcifications that originated from BAC but were of minor extent were categorized as BAC Grade 3, while BAC Grade 4 indicated pronounced BAC. In cases with multiple BAC foci, the highest grade observed was recorded.

### 2.4. Reader Protocol

One radiologist (J.A.S., 4 years of experience) independently evaluated all mammograms. A random sample comprising 20% of all included patients was independently evaluated by a second (J.H., 3 years of experience) and a third blinded reader (C.M., 10 years of experience).

### 2.5. Statistical Analysis

All statistical analyses were performed using R (Version 4.3.2, R Foundation for Statistical Computing, Vienna, Austria). Continuous variables are reported as medians with interquartile range (IQR) or mean with standard deviation (SD), as appropriate. Categorical variables are presented as absolute frequencies and percentages. Group comparisons were conducted using the Wilcoxon rank sum test, Welch’s two-sample *t*-test, Pearson’s Chi-square test, or Fisher’s exact test, depending on data distribution and sample size. To compare the prevalence of BAC between lung transplant recipients and controls at baseline, Fisher’s exact test or Chi-square tests were applied. Baseline characteristics, including age, smoking status, diabetes mellitus, hypertension, and chronic kidney disease (CKD), were evaluated for significant group differences. Multivariable logistic regression was further used to assess associations between lung transplantation and the presence of BAC, grouped microcalcifications, and macrocalcifications at the last mammography, adjusting for age, CKD, diabetes mellitus, and arterial hypertension. Odds ratios (ORs) with 95% confidence intervals (CIs) and two-sided *p*-values were reported. The progression of BAC and other mammographic findings was analyzed in patients with serial imaging. Annual progression rates were summarized using violin plots. Cumulative incidence curves with log-rank testing were used to evaluate time to mammographic findings (e.g., BAC progression, macrocalcifications, grouped microcalcifications, BI-RADS ≥ 4 lesions). A two-sided *p*-value < 0.05 was considered statistically significant. Interobserver agreement for BAC scoring was assessed using Fleiss’ Kappa, with interpretation according to Landis and Koch: values ≤ 0.20 indicated poor agreement; 0.21–0.40, fair; 0.41–0.60, moderate; 0.61–0.80, substantial; and 0.81–1.00, excellent agreement.

## 3. Results

### 3.1. Patient Cohort

**Figure 1:**
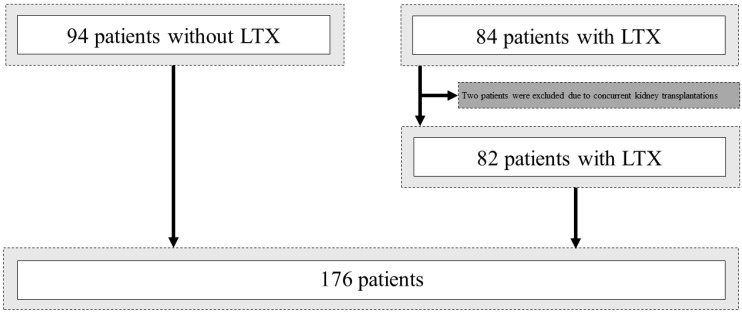
Flowchart illustrating inclusion and exclusion. LTX, lung transplantation.

A total of 176 patients were included in the study, comprising 94 controls and 82 lung transplant (LTX) recipients (Figure 1).

The median age at first mammography was 56 years (IQR 50–61), with no significant difference between groups (*p* = 0.71). Among LTX recipients, the most common underlying lung disease was chronic obstructive pulmonary disease (COPD), present in 43.9% of cases, followed by cystic fibrosis (14.6%), idiopathic pulmonary fibrosis and lymphangioleiomyomatosis (each 7.3%), alpha-1 antitrypsin deficiency (6.1%), and pulmonary arterial hypertension (3.7%). In approximately 5% of cases, the underlying lung disease was unclassified or represented overlapping conditions. Other diagnoses, including non-CF bronchiectasis, sarcoidosis, and interstitial lung diseases, were identified in fewer than 2% of patients. All patients who underwent LTX received immunosuppressive therapy consisting of a combination of agents, including a calcineurin inhibitor (cyclosporine or tacrolimus), antiproliferative agents (azathioprine, mycophenolate, sirolimus, or everolimus), and corticosteroids. LTX recipients were significantly more likely to be current or former smokers (38% vs. 9.6%; *p* < 0.001) and had a higher mean pack-year exposure (13.4 ± 19.4 vs. 2.4 ± 8.0; *p* < 0.001) compared to controls. Chronic kidney disease was more common in the LTX group (59% vs. 8.5%; *p* < 0.001), whereas the prevalence of diabetes mellitus (8.5% vs. 15%; *p* = 0.22) and arterial hypertension (38% vs. 27%; *p* = 0.14) did not differ significantly between groups. Pulmonary arterial hypertension was observed exclusively in the LTX group (7.3%; *p* = 0.009). Baseline characteristics are summarized in Table 1.

### 3.2. Imaging Characteristics and Follow-Up

The number of mammographies per patient was significantly lower in the LTX group, with a median of 2 (IQR 1–4), compared to 4 (IQR 3–6) in controls (*p* < 0.001). The median follow-up duration was also shorter among LTX recipients (4.68 years, IQR 2.17–8.02) than in controls (9.01 years, IQR 5.05–11.69; *p* < 0.001). Additionally, only 45% of LTX patients underwent three or more mammographies, compared to 82% of controls (*p* < 0.001). An overview is provided in Table 2.

### 3.3. Mammographic Assessment and BAC Scoring

At first mammography, breast density distribution in the LTX group was: A: 9.8% (8 of 82), B: 24% (20 of 82), C: 37% (30 of 82), and D: 29% (24 of 82) (Table 3). In the control group, corresponding proportions were: A: 14% (13 of 94), B: 34% (32 of 94), C: 37% (35 of 94), and D: 15% (14 of 94). Notably, at last mammography, category D density remained more prevalent in LTX recipients compared to controls (15% vs. 4.3%; *p* = 0.008). Most mammographies were classified as BI-RADS 2 at both first (LTX: 87%, Control: 85%) and last imaging (LTX: 89%, Control: 89%). Grouped microcalcifications were significantly more common in the LTX group at last mammography (21% vs. 3.2%; *p* < 0.001), while macrocalcifications or sporadic scattered microcalcifications were observed in 80% of LTX recipients and 70% of controls (*p* = 0.12). BAC were markedly more frequent and pronounced in LTX patients. BAC was absent in 60% of LTX recipients compared to 89% of controls (*p* < 0.001).

### 3.4. Multivariable Analysis

Multivariable logistic regression analysis (Table 4) showed that lung transplantation was independently associated with grouped microcalcifications (OR 9.26, 95% CI 2.29–50.4; *p* = 0.004) and breast arterial calcifications (BAC; OR 8.15, 95% CI 2.77–27.0; *p* < 0.001). Diabetes mellitus was additionally associated with grouped microcalcifications (OR 5.33, 95% CI 1.15–24.7; *p* = 0.029), and arterial hypertension with BAC (OR 3.05, 95% CI 1.31–7.25; *p* = 0.010). No significant associations were observed for macrocalcifications.

### 3.5. Progression Analysis and Cumulative Incidence Curves

**Figure 2:**
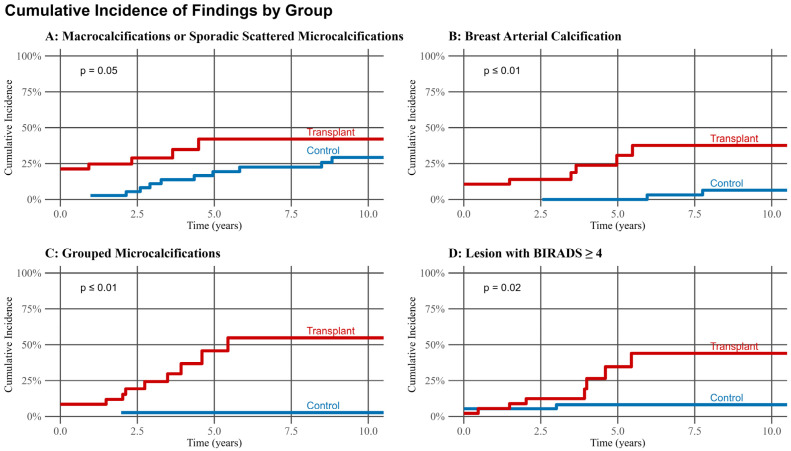
Cumulative incidence of mammographic findings over the first 10-years of the follow-up period of 94 patients in the control group and 82 patients in the lung transplantation group.

**Figure 3:**
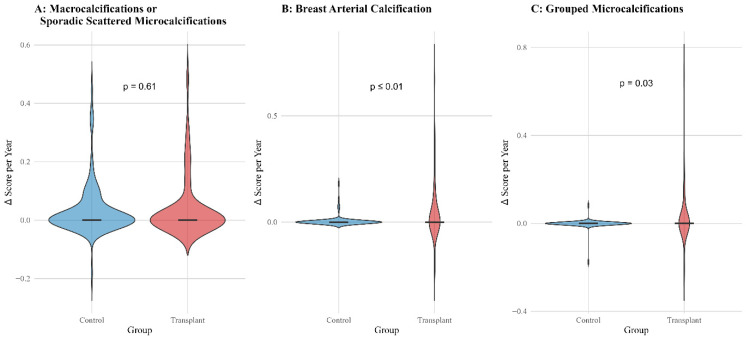
Annual progression rates of mammographic findings of 94 patients in the control group and 82 patients in the Lung transplantation group by group. Violin plots illustrate the distribution of annual score changes (Δ Score per Year) for different mammographic findings in transplant recipients (red) and controls (blue).

Cumulative incidence analysis (Figure 2) revealed a consistently higher incidence of all evaluated mammographic findings in transplant recipients compared to controls over the 10-year follow-up period (all *p* ≤ 0.01). This included macrocalcifications or sporadic scattered microcalcifications, BAC, grouped microcalcifications, and BI-RADS ≥ 4 lesions. Annual progression rates (Figure 3) underscored these findings: while no significant difference was observed in the progression of macrocalcifications (*p* = 0.61), LTX recipients exhibited significantly faster progression of both BAC (*p* ≤ 0.01) and grouped microcalcifications (*p* = 0.03). Biopsy procedures were performed in 6 patients (6.4%) in the control group and 8 patients (9.8%) in the LTX group, with no significant difference in biopsy frequency or method (*p* = 0.69). In the control group, four patients underwent core needle biopsy and two underwent tomosynthesis-guided biopsy; in the LTX group, five underwent core needle biopsy and three underwent tomosynthesis-guided biopsy. Ductal carcinoma in situ (DCIS) was diagnosed in four patients (4.3%) in the control group and none of the LTX recipients (*p* = 0.12). Invasive breast cancer was observed in three patients in each group (3.2% in control, 3.7% in LTX), with no significant difference between groups (*p* = 1.00).

### 3.6. Interobserver Agreement

Fleiss’ kappa analysis demonstrated varying levels of inter-rater agreement across the evaluated calcification categories. Agreement was moderate for both macrocalcifications (κ = 0.51) and the presence of BAC (κ = 0.59). Grouped microcalcifications showed fair agreement (κ = 0.33), highlighting the interpretive challenges associated with this subtype. Notably, the 4-level ordinal BAC scoring system achieved the highest concordance, with substantial agreement among the three readers (κ = 0.61).

## 4. Discussion

In this study, we investigated the incidence and progression of grouped microcalcifications, macrocalcifications or sporadic scattered microcalcifications and breast arterial calcification in lung transplant recipients. Our findings indicate that grouped microcalcifications and BAC are significantly more prevalent in lung transplant recipients compared to healthy individuals, and that their progression occurs at an accelerated rate in this population, potentially complicating radiologic differentiation between benign vascular calcifications and malignant-appearing microcalcifications.

To our knowledge, no comparable data exist on the prevalence of grouped microcalcifications and BAC in lung transplant recipients. Our data shows a higher prevalence of BAC in lung transplant recipients compared to controls, while the underlying mechanisms remain unclear. BAC typically involves the middle layer of the mammary arteries [19]. The literature reports a high prevalence among patients with chronic kidney disease (CKD), diabetes mellitus, hypertension and after kidney transplantation [23,24,25]. While we could confirm the association of diabetes mellitus and hypertension with BAC our data interestingly did not show an association with CKD. Other risk factors for developing BAC include age, hypertension, and hormonal changes, particularly post-menopause [26]. While smoking is a well-established risk factor for vascular calcification and is associated with BAC, many lung transplant recipients, particularly those with cystic fibrosis, are lifelong non-smokers, yet still show pronounced BAC [27]. This suggests that alternative factors, such as chronic inflammation or immunosuppressive medication, may be driving the calcification process. Chronic pulmonary disease is associated with systemic inflammation, and immunosuppressive agents used post-transplant are known to contribute to vascular remodeling and endothelial dysfunction [28,29]. Moreover, atherosclerosis itself is increasingly understood as an inflammatory condition, supporting the hypothesis that BAC in this population reflects a transplant-specific inflammatory vasculopathy [30].

**Figure 4:**
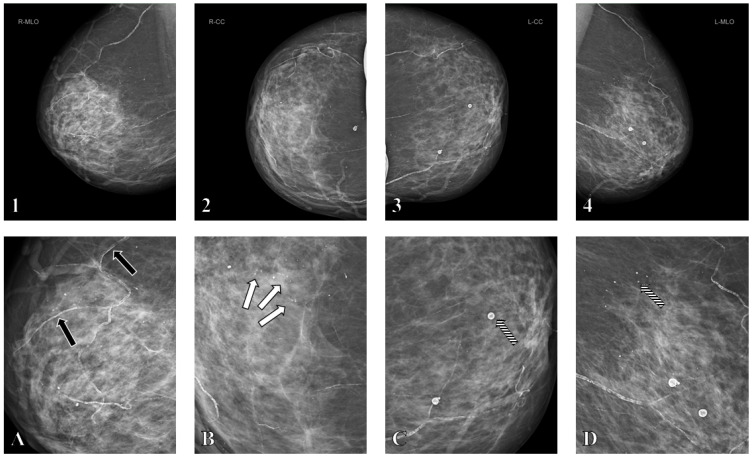
Screening mammography in a female patient in her late fifties approximately 8 years after bilateral lung transplantation. Mammography demonstrates diffuse breast calcifications including partly grouped microcalcifications, macrocalcifications, and breast arterial calcifications (BAC) in the right breast in mediolateral oblique (**1**) and craniocaudal (**2**) projections, and the left breast in mediolateral oblique (**3**) and craniocaudal (**4**) projections. Magnification images (**A**–**D**) highlight the different types of breast calcifications, including grouped microcalcifications ((**B**), white arrows), macrocalcifications ((**C**,**D**), striped arrows), and BAC ((**A**), black arrows). Due to the presence of diffuse microcalcifications, these prominent grouped microcalcifications were assigned BI-RADS 3 rather than BI-RADS 4, and follow-up mammography after 6 months was recommended instead of tomosynthesis-guided biopsy. Follow-up imaging showed no progression.

Accurate differentiation of microcalcifications on mammography is essential for effective patient management. BAC can closely mimic malignant calcifications, and several case reports describe biopsies of BAC due to its resemblance to breast cancer [14,31]. This overlap is particularly problematic in early-stage BAC, which can resemble grouped microcalcifications, a feature present in up to 40% of breast cancers [10,18,20]. The challenge becomes even greater in lung transplant recipients, who exhibit more frequent grouped microcalcifications and BAC than controls, especially when isolated calcifications lack tubular morphology amid extensive vascular, micro, and macrocalcifications, as shown in Figure 4 [26,32].

Although suspicious findings were more frequent in the transplant group and led to an increased number of biopsies, the incidence of DCIS remained comparable between groups, underscoring the clinical challenge of accurately distinguishing benign from malignant calcifications in this complex population. To avoid unnecessary biopsies radiologists should pay close attention to BAC, particularly when calcifications appear punctate, short linear, or suspiciously mimic vascular patterns, and may consider using a BAC severity score. The BI-RADS 5th edition lexicon defines suspicious calcifications as amorphous, coarse heterogeneous, fine pleomorphic, or fine linear. Maxwell et al. proposed a five-tier system (M1–M5) to stratify risk, while the ACR BI-RADS categories range from 1 (no findings) to 5 (highly suspicious) [33,34]. Saenger et al. recently introduced a BAC-specific four-grade visual scale, from no or scattered findings (Grade 1) to pronounced BAC (Grade 4) [20]. Such scoring systems may help focus attention on truly suspicious microcalcifications and reduce unnecessary biopsies. If uncertainty remains and tomosynthesis is available, supplemental digital breast tomosynthesis may aid in distinguishing BAC from malignant calcifications [35]. In the future, artificial intelligence could support this differentiation through automated detection and classification [36].

Cancer has emerged as a leading cause of mortality in transplant recipients, accounting for 13–14% of deaths between 5 and 10 years post-transplantation, with a study by Acuna et al. reporting an increased risk of all cancer deaths by almost threefold in solid organ transplant recipients [37,38,39,40]. In lung transplant recipients studies have reported a 3.7 to 5.6-fold higher incidence of cancer relative to the general population [41,42]. While the overall risk of malignancy steadily increases with time after LTX, this trend does not appear to extend to breast cancer, whose incidence remains similar to that of the general population [43,44,45,46]. In our cohort, DCIS was detected in 4.3% of controls but in none of the LTX recipients, while invasive breast cancer occurred at a similar rate in both groups (3.2% vs. 3.7%). Thus, despite the increased prevalence of calcifications in LTX recipients, this did not translate into a higher breast cancer detection rate, and no oncological benefit of calcification assessment was demonstrated. Consistent with existing literature, we also did not observe an increased incidence of breast cancer in LTX recipients compared to the general screening population. Data regarding the effect of immunosuppressive therapy on the cancer risk remain conflicting [7,21,47,48,49]. Research indicates that transplant recipients may experience more aggressive malignancies, face poorer outcomes, with at least double the breast cancer-related mortality risk, younger age at diagnosis, and more advanced disease stages [7,37,50,51]. Due to substantial heterogeneity in treatment regimens of immunosuppressive therapy, variable dosages, and frequent modifications during follow-up, often occurring between mammographic examinations, the specific impact of immunosuppressive therapy on our findings could not be reliably assessed.

Breast cancer screening in lung transplant recipients poses unique ethical and clinical challenges. All female candidates undergo mandatory pre-transplant mammography, as any suspicion of malignancy precludes transplantation. As a result, even BI-RADS 3 findings, which would typically warrant follow-up in two years, must be fully worked up, since many patients may not survive such a delay. Despite this, current screening practices still follow general population guidelines [40,52]. While organized screening programs reduce breast cancer mortality by 25–40% in women aged 50–70, there are no specific recommendations for lung transplant recipients [2,53,54]. Data on screening efficacy in this group are lacking [4,7]. Evidence from kidney transplant cohorts suggests that life-year gains from screening are over 50% lower than in the general population, raising concerns about overdiagnosis and overtreatment in patients with limited life expectancy [55]. Therefore in lung transplant patients, the utility of breast cancer screening necessitates careful ethical consideration due to limited life expectancy and the increased potential for overdiagnosis and overtreatment [56]. Future guidelines should weigh the trade-off between early detection and potential harm from overdiagnosis in this high-risk but vulnerable population.

Several limitations must be considered when interpreting the results of this study. First, the retrospective design may have introduced confounding and selection bias. Including only patients with ≥2 mammograms likely favored healthier survivors, potentially underestimating calcification prevalence in patients with shorter follow-up. Second, a major limitation is the absence of a control group with advanced chronic lung disease, to clarify the role of underlying lung disease versus transplantation. This was not feasible due to limited survival and lack of serial mammographic data in such patients.

Third, as a single-center study conducted at a tertiary referral hospital, the findings may not be generalizable to broader populations or different clinical settings. Fourth, the incomplete availability of longitudinal clinical data limited the accuracy of comorbidity assessment. Medical histories were based on the last available report and assumed to remain constant over time, which may have resulted in over- or underestimation of their influence. Fifth, heterogeneity in mammography protocols, equipment, and radiologist experience across the 12-year study period could have introduced variability despite standardized assessment criteria. Sixth, digital breast tomosynthesis was not routinely available and was excluded from the analysis, potentially limiting the ability to resolve overlapping calcifications. Seventh, histopathologic confirmation was not available for all suspicious findings, and we cannot exclude the possibility of undiagnosed or misclassified malignancies, particularly in patients managed conservatively.

In conclusion, our study shows that lung transplant recipients exhibit a significantly higher prevalence and faster progression of both BAC and grouped microcalcifications compared to matched controls. These findings underscore the diagnostic challenges posed by vascular and non-malignant calcifications in this high-risk population. As BAC can mimic suspicious microcalcifications, particularly in fragmented or atypical forms, radiologists might consider employing standardized BAC scoring systems to enhance diagnostic accuracy. Future screening strategies may benefit from the integration of artificial intelligence and tailored protocols to minimize unnecessary biopsies while ensuring early cancer detection.

## Figures and Tables

**Table 1:** Baseline characteristics of the study population by group (Control vs. LTX).

Characteristic	Overall, N = 176	Control, N = 94	LTX, N = 82	*p*-Value
Age at first mammography	56 (50, 61)	55 (51, 61)	56 (50, 62)	0.7 ^1^
Current or former smoker	40 (23)	9 (9.6)	31 (38)	<0.001 ^2^
Pack years, Mean (SD)	7.5 (15.4)	2.4 (8.0)	13.4 (19.4)	<0.001 ^3^
Chronic Kidney Disease	56 (32)	8 (8.5)	48 (59)	<0.001 ^2^
Diabetes mellitus	21 (12)	14 (15)	7 (8.5)	0.2 ^2^
Arterial Hypertension	56 (32)	25 (27)	31 (38)	0.14 ^2^
Pulmonary Arterial Hypertension	6 (3.4)	0 (0)	6 (7.3)	0.009 ^2^

Median (Q1, Q3) or N (%) are shown unless otherwise specified. Some percentages may not sum to 100% due to rounding. LTX, lung transplantation; SD, standard deviation. ^1^ Wilcoxon rank sum test. ^2^ Fisher’s Exact Test. ^3^ Welch Two-Sample *t*-test.

**Table 2:** Imaging Characteristics, Number of Mammographies, and Follow-up in Control and Lung Transplant Groups.

Characteristic	Overall, N = 176	Control, N = 94	LTX, N = 82	*p*-Value ^1^
Number of available mammographies	3.00 (2.00, 5.00)	4.00 (3.00, 6.00)	2.00 (1.00, 4.00)	<0.001
Interval between first and last mammography	6.85 (3.37, 10.47)	9.01 (5.05, 11.69)	4.68 (2.17, 8.02)	<0.001
Patients with ≥3 mammograms	114 (64)	77 (82)	37 (45)	<0.001
Follow-up, years	6.95 (3.37, 10.47)	9.01 (5.05, 11.69)	4.68 (2.17, 8.02)	<0.001

Median (Q1, Q3) or N (%) are shown unless otherwise specified. Some percentages may not sum to 100% due to rounding.; LTX, lung transplantation. ^1^ Fisher’s Exact Test.

**Table 3:** Comparison of Mammographic Findings between Lung Transplant Recipients and Controls at First and Last Available Mammography.

Characteristic	First Available Mammography			Last Available Mammography		
	No LTX, N = 94	LTX, N = 82	*p*-Value	No LTX, N = 94	LTX, N = 82	*p*-Value
ACR			0.10 ^1^			0.008 ^1^
a	13 (14)	8 (9.8)		17 (18)	6 (7.3)	
b	32 (34)	20 (24)		39 (41)	25 (30)	
c	35 (37)	30 (37)		34 (36)	39 (48)	
d	14 (15)	24 (29)		4 (4.3)	12 (15)	
BIRADs, n (%)			0.3 ^1^			0.2 ^1^
0	0 (0)	1 (1.2)		0 (0)	0 (0)	
1	8 (8.5)	2 (2.4)		4 (4.3)	0 (0)	
2	80 (85)	71 (87)		84 (89)	73 (89)	
3	3 (3.2)	4 (4.9)		4 (4.3)	6 (7.3)	
4	3 (3.2)	4 (4.9)		1 (1.1)	3 (3.7)	
5	0 (0)	0 (0)		1 (1.1)	0 (0)	
6	0 (0)	0 (0)		0 (0)	0 (0)	
Grouped microcalcifications	3 (3.2)	5 (6.1)	0.5 ^2^	3 (3.2)	17 (21)	<0.001 ^2^
Macrocalcifications or sporadic, scattered microcalcifications	45 (48)	44 (54)	0.4 ^2^	66 (70)	66 (80)	0.12 ^2^
BAC score			0.003 ^1^			<0.001 ^1^
1	89 (95)	67 (82)		84 (89)	49 (60)	
2	4 (4.3)	3 (3.7)		4 (4.3)	4 (4.9)	
3	1 (1.1)	9 (11)		6 (6.4)	14 (17)	
4	0 (0)	3 (3.7)		0 (0)	15 (18)	

N (%) are shown unless otherwise specified. Some percentages may not sum to 100% due to rounding. BAC, Breast Arterial Calcifications; LTX, lung transplantation. ^1^ Pearson’s Chi-squared test. ^2^ Fisher’s Exact Test.

**Table 4:** Multivariable logistic regression analysis for the presence of mammographic calcifications at last mammography.

Characteristic	Macrocalcifications or Sporadic Scattered Microcalcifications	Grouped Microcalcifications	BAC
Lung transplantation	1.37 (0.55–3.52), 0.50	9.26 (2.29–50.4), 0.004	8.15 (2.77–27.0), <0.001
Age (years)	1.00 (0.96–1.05), >0.90	0.93 (0.87–1.00), 0.051	0.99 (0.94–1.04), 0.70
Chronic kidney disease	1.49 (0.56–4.15), 0.40	0.48 (0.15–1.49), 0.20	1.41 (0.57–3.48), 0.50
Diabetes mellitus	2.97 (0.76–19.7), 0.20	5.33 (1.15–24.7), 0.029	2.33 (0.68–7.97), 0.20
Arterial hypertension	2.04 (0.88–5.20), 0.11	1.65 (0.53–5.04), 0.40	3.05 (1.31–7.25), 0.010
Smoking status	1.18 (0.47–3.20), 0.70	3.12 (0.94–10.8), 0.064	0.68 (0.25–1.77), 0.40

OR (95% CI), *p* is shown unless otherwise specified. BAC, Breast Arterial Calcifications; OR, odds ratio; CI, confidence interval.

## Data Availability

The data supporting this study are not available due to ethical considerations and institutional policy.

## Abbreviations

BAC	breast arterial calcifications
BI-RADS	Breast Imaging Reporting and Data System
DCIS	ductal carcinoma in situ
LTX	Lung transplant recipients
